# Preparation and Physico-Chemical Performance Optimization of Sintering-Free Lightweight Aggregates with High Proportions of Red Mud

**DOI:** 10.3390/ma14010218

**Published:** 2021-01-05

**Authors:** Guan Wang, Chao Zhang, Wenlong Wang, Shuang Wu, Jingwei Li, Xujiang Wang, Changliang Wu

**Affiliations:** National Engineering Laboratory for Reducing Emissions from Coal Combustion, Engineering Research Center of Environmental Thermal Technology of Ministry of Education, Shandong Key Laboratory of Energy Carbon Reduction and Resource Utilization, School of Energy and Power Engineering, Shandong University, Jinan 250061, China; wangguan2307@163.com (G.W.); zc17861411977@163.com (C.Z.); wushuanglc@163.com (S.W.); ljw@sdu.edu.cn (J.L.); x.wang@sdu.edu.cn (X.W.); maomaochong_wcl@163.com (C.W.)

**Keywords:** red mud, binder material, red mud based on sintering-free lightweight aggregates, physico-chemical performance, heavy metal leaching

## Abstract

Sintering-free lightweight aggregates were prepared with high proportions of red mud and a binder material derived from whole solid wastes through rolling granulation at room temperature. The preparation process was optimized by changing the material matching and size parameters of the SFLAs. The physico-chemical performance, including the density, mechanical strength, water absorption, hydration products, heavy metal leaching, and microstructure were evaluated by jointly employing X-ray Fluorescence, X-ray Diffraction, and Inductively Coupled Plasma Optical Emission Spectrometry, Shadow Electron Microscope, etc. The results indicated that the red mud and waste-based binders were highly compatible in the granulation process, with up to 80% red mud being successfully added. The sintering-free lightweight aggregates products at the binder content of 30% and the size coverage of 10–16 mm exhibited a bulk density of 900–1000 kg·m^−3^, a 28 d cylinder compressive strength of 9.2–11.3 MPa, and water absorption of less than 10%. Owing to the formation of important hydration products, ettringite, the heavy metal leaching of the sintering-free lightweight aggregates was also proven to be environmentally acceptable. This work provides a promising pathway to prepare low-cost, high-strength, and green lightweight aggregates through the large-scale utilization of solid waste red mud.

## 1. Introduction

Red mud (RM) is an industrial solid waste produced during alumina production by refining bauxite [1]. Generally, the Bayer process, Bayer-sintering combination process, and sintering process are applied, where the Bayer process is used in 95% of alumina production owing to the low energy consumption and simple production process [2]. The production of 1 t of alumina typically generates 0.8–1.8 t of RM, and a large quantity of RM (about 120 million t) is generated annually on a global scale, especially 40% of which is generated in China [3,4,5]. At present, there are no defined methods for the disposal of RM, and this material is generally disposed of on the ground, thereby consuming vast tracts of usable land [6]. Furthermore, the highly alkaline (pH 10–12.5) discharged RM causes serious environmental problems such as soil contamination, land basification, and water pollution [7,8]. Therefore, it is urgent to explore the comprehensive utilization of red mud to reduce the environmental damage caused by its storage.

Since the 1950s, considerable research and development have been devoted to the utilization of RM globally [9]. Considering the abundance of valuable components, including Fe, Al, Ti, and rare earth elements, RM is considered a valuable secondary resource [10]. Although a lot of work and research has been done on the recovery and utilization of the valuable components of RM through hydrometallurgical and pyrometallurgical methods in recent years, the unique physical and chemical properties of RM prevent large-scale utilization of RM based on these methods. At present, the treatment and utilization of RM are concentrated on the manufacture of adsorbents, ceramics, bricks, high-performance concrete admixtures, and base materials for roads [11,12,13,14,15]. Despite extensive previous studies on how to utilize RM comprehensively on a large-scale, most of the processes for the utilization of RM are limited by high cost and high energy consumption; thus, studies are still at the laboratory stage and no real engineering application has been realized [16]. Therefore, to realize the comprehensive utilization of RM, innovative techniques for the low-cost and low-energy consumption of RM must be developed.

The production of sintering-free lightweight aggregates (SFLAs) (also known as cold-bonded lightweight aggregates) is widely used to recycle solid wastes by the cold-bonded pelletizing technique [17], where the choice of raw materials for SFLAs has been extended to various solid wastes in recent years, including waste water treatment sludge [18], ground granulated blast furnace slag [19], municipal solid waste incinerator fly ash [20], and water paper sludge ash [21]. The widespread research on the production SFLAs using solid wastes may be proposed as a feasible method on the exploration of producing SFLAs using RM, which has been rarely studied. Besides, RM itself is characterized by low density, porosity, and high viscosity, etc. [22], and thus can be used as a dominant raw material in the production of SFLAs. In this manner, the integral target of immobilizing waste RM in cementitious materials by producing SFLAs that can be used in concrete while converting the RM from waste to a value-added material on a large-scale.

Cementitious binders are an essential element in the production of SFLAs, especially when raw materials with little or no cementitious properties are used. Cement is the conventional binder used in the literature [23]. Besides cement, bentonite, glass powder, lime, and clay binders have also been utilized as binders for conventional lightweight aggregate (LAs) production [24,25]. However, the property and cost constraints have prompted researchers to employ alternative binders. Therefore, some solid wastes with potential activity and an alkaline activator were utilized to produce SFLAs in previous studies. Although this preparation method makes up the lack of energy consumption of sintering lightweight aggregate, the variety of solid wastes that can be utilized is limited to potential activity solid wastes. However, using typical RM with no cementitious properties produced a large-scale SFLAs, a new alternative binder with low-cost and high properties must be developed. At the same time, the SFLAs prepared are environmentally friendly and do not cause secondary environmental pollution. If this is achieved, SFLAs production using RM can become feasible.

Hence, in this study, a new concept for SFLAs preparation using RM combined with solid waste-based binder materials was presented. The solid waste-based binder materials is synthesized according to the method presented in a previous study by using red mud, carbide slag, aluminum ash, and flue gas desulfurization gypsum (FGD gypsum) [26]. The preparation cost is low, and the materials have excellent features of early strength, high strength, and fast hardening. A pelletizing disk is used to manufacture the RM-based SFLAs. After preparation, the properties of RM-based SFLAs were tested. Additionally, the leaching behavior of the SFLAs was tested and examined according to the related standards. Herein, the feasibility of SFLAs production was reported by using RM combined with solid waste-based binder materials.

## 2. Experimental

### 2.1. Raw Materials and Characterization

As the main raw material, RM was collected from Xinfa Conglomerate (Shandong, China), which was produced from the Bayer process. The basic physical properties of Bayer RM were shown in Table 1. The main chemical components of the RM are Fe_2_O_3_, Al_2_O_3_, and SiO_2_, and the mineral composition mainly comprises hematite, anorthite, calcite, and quartz. The binder materials were prepared from industrial solid wastes (aluminum dust, FGD gypsum, carbide slag, and RM). FGD gypsum was supplied by Liaocheng Coal-fired Power Plants (Shandong, China), its main chemical components are CaO and SO_3_, and the main mineral is calcium sulfate dihydrate (CaSO_4_·2H_2_O). Aluminum dust was supplied by the Xinfa Group (Shandong, China). Carbide slag was obtained from Liaocheng Acetylene Company (Shandong, China). The aluminum dust and carbide slag contained high levels of Al_2_O_3_ and CaO, the main mineral phases of aluminum dust are aluminum (Al) and aluminum oxide (Al_2_O_3_). Calcium carbide slag was the waste residue of hydrolysis of calcium carbide, and calcium hydroxide (Ca(OH)_2_) is its main chemical component.

**Figure 1:**
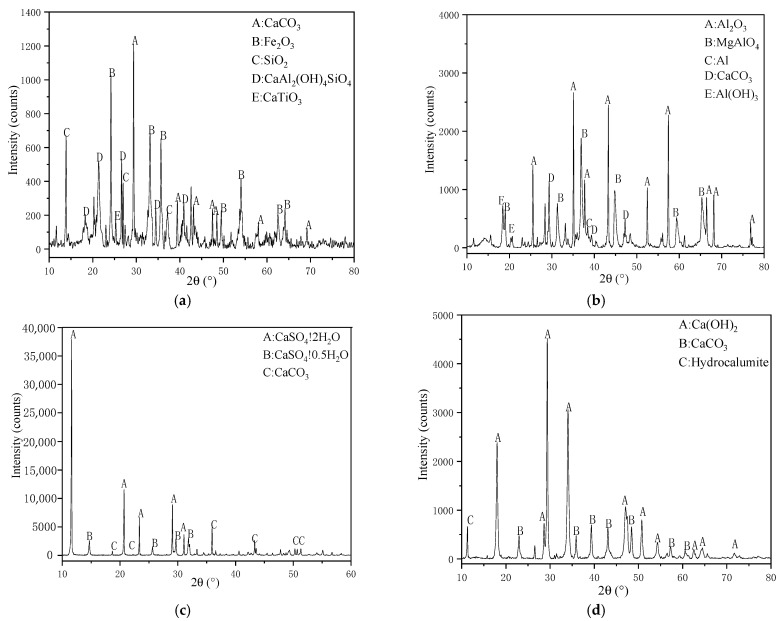
XRD pattern of raw materials (**a**): red mud (RM), (**b**): FGD gypsum, (**c**): aluminum dust, (**d**): carbide slag.

The raw materials were placed into a 105 °C drying box to achieve constant weight and set aside. An X-ray fluorescence spectrometer (XRF; SPRCTRD Analytical Instruments GmbH, Kleve, Germany) was used to determine the chemical composition of the raw materials. X-ray diffraction (XRD; PANalytical B.V., Almelo, Netherlands) was used to detect the mineral phase composition of the raw materials. The test conditions were as follows: a copper target X-ray tube was used at a tube current of 30 mA; the tube voltage was 40 kV; the continuous scanning mode was adopted; the scanning range was 10–80°, and the step length was 0.02°. Table 2 shows the chemical composition of the different raw materials, and Figure 1 shows the XRD patterns of these raw materials.

### 2.2. Preparation of Solid Waste-Based Binder

**Figure 2:**
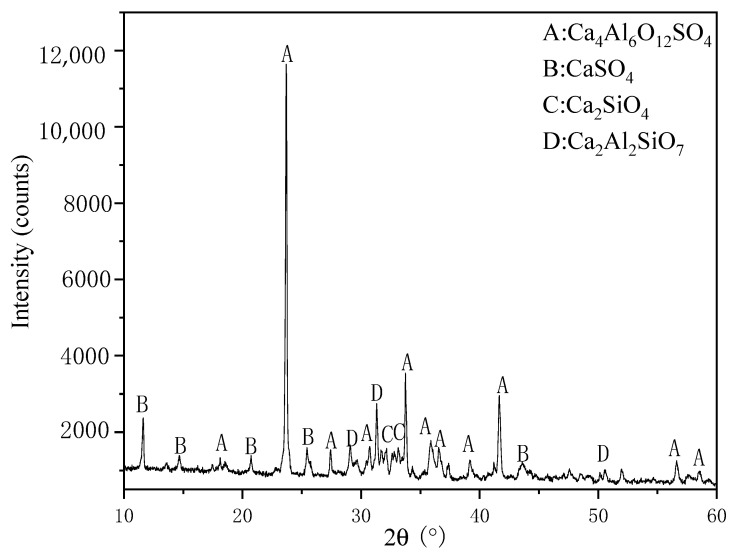
XRD pattern of binder materials.

As shown in Table 2, RM contains Fe_2_O_3_, Al_2_O_3_, and SiO_2_. The main chemical components of FGD gypsum are CaSO_4_. Aluminum dust and calcium carbide slag contain high levels of Al_2_O_3_ and CaO, respectively. The solid waste-based sulphoaluminate cement clinker was synthesized by the synergistic combination of four kinds of industrial solid wastes (RM, aluminum ash, FGD gypsum, and carbide slag). At present, our laboratory has fully mastered the preparation technology of these binder materials, and has realized industrialized production. The preparation method and specific control parameters were set as follows: the alkalinity coefficient was 0.95–1.05, the aluminum-sulfur ratio was P = 1.9, and the aluminum-silicon ratio was N = 3.4 [27]. By controlling the alkalinity coefficient and adjusting the ratio of raw materials, as shown in Table 3, the solid waste-based sulphoaluminate cement clinker was obtained after calcining at 1250 °C, and heating preservation time for 45 min [28]. Finally, 8% FGD gypsum was added to obtain the solid waste-based sulphoaluminate cement [29], after grinding and mixing, the solid waste-based sulphoaluminate cement was obtained. The compressive strength of solid waste-based sulphoaluminate cement prepared by different ratios were tested, respectively. When the alkalinity coefficient was 0.90, the sulphoaluminate cement prepared had the best performance and it was used as the best proportion of the raw material to prepare solid waste-based sulphoaluminate cement. The solid waste-based sulphoaluminate cement prepared by the best proportion of the raw material was used as the binder materials for the preparation of RM based on SFLAs, it is hereinafter referred to as the binder materials. Table 4 shows the chemical composition of these binder materials, and Figure 2 shows the XRD pattern of these binder materials.

### 2.3. Preparation of RM-Based SFLAs

**Figure 3:**
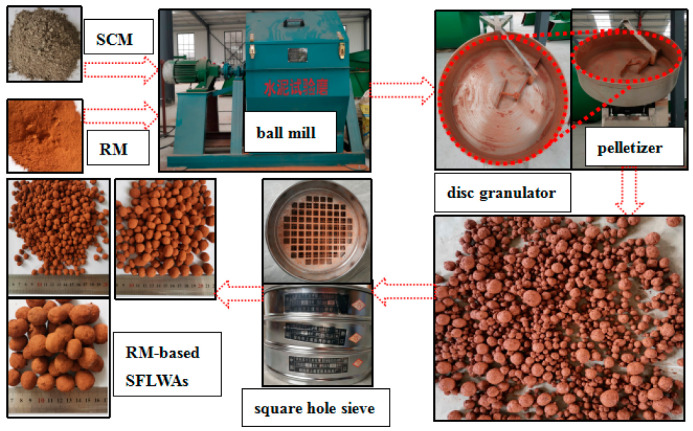
Preparation process of RM-based sintering-free lightweight aggregates (SFLAs) through cold bonded granulation used in this work.

ZL-5 disc granulator was used to manufacture RM-based SFLAs through cold bonded granulation. These solid waste-based binder materials were used in the production of RM-based SFLAs. The preparation process parameters of RM based on SFLAs are shown in Table 5, which includes the diameter of the pelletizing disc, the inclination angle of the pelletizing disc, and critical revolutions of the pelletizing disc. RM and binder were dry for one hour at 105 °C in a drying box, the dry material was ground and mixed well according to the preset ratio (Table 5) in a small ball mill. The prepared materials are evenly distributed in the pelletizing disc, water is added to the pelletizing disc in the form of spraying, the powder becomes spherical aggregates under the effect of cementing generated by binder materials hydration reaction and centrifugal force produced by the rotation of pelletizing, which ultimately got collected from pelletizing disc, RM-based SFLAs were obtained after drying and sieving, which was naturally cured at room temperature (20 °C) until they were tested for different properties. Figure 3 shows the complete preparation process of RM-based SFLAs and equipment.

### 2.4. Characterization of Solid Waste-Based Binder Materials and RM-Based SFLAs

#### 2.4.1. Performance Test of Solid Waste-Based Binder Materials

The compressive strength, flexural strength, and setting time of the binder materials were tested according to the requirements of GB/T 20472-2006 [30]. Mortar test blocks were prepared with the water to binder materials and quartz sand to binder materials ratios of 0.45 and 3, respectively, mix evenly in the mixer, mortar was molded into 40 mm × 40 mm × 160 mm in a steel mold. The molded mortar was demolded after 6 h and cured in a moist cabinet at 20 °C and 95% R.H. until 1 d, 3 d, and 28 d, respectively. According to the GB/T 17671-1999 [31] “Cement Mortar Strength Inspection Method (ISO)”. the compressive strength, flexural strength, and setting time of Portland cement were tested. Mortar test blocks of Portland cement were prepared with the water to cement ratios of 0.5, other test methods were the same as above. The compressive and flexural strength of the mortar was tested by using a DYE-2000 digital pressure testing machine. The reported results were the average of six mortars.

#### 2.4.2. Performance Test of RM-Based SFLAs

According to GB/T 17431.2-2010 [32] “Lightweight Aggregate and Test Methods”, the bulk density, water absorption, apparent density, and cylinder compressive strength of all the samples were determined.

Bulking density test: take the 3 L dry samples, and pour the sample evenly into the standard measuring cylinder with a material shovel, the upper part of the measuring cylinder is filled with samples into a cone, then use a ruler to smooth the sample along the edge of the measuring cylinder from the center to both sides, the surface depression was filled with smaller particle size SFLAs, weighed and recorded as *m*_1_. The heap density was calculated according to Equation (1), and the calculation accuracy is 1%.

(1) $$\rho_{b}=\frac{\left(m_{1}-m_{0}\right)\times 1000}{V_{1}}$$

where *ρ*_b_ is the bulk density (kg/m^3^); *m*_1_ is the total mass of the sample and the measuring cylinder (kg), *m*_0_ is the mass of the measuring cylinder (kg); *V*_1_ is the volume of the measuring cylinder (m^3^).

Water absorption test: take the 1–2 L dry samples, weigh them, and write it as *m*_3_, then put the samples into the water to soak them for 1 h, and then take them out. Pour them into a 2.36 mm sieve to filter water for 1–2 min, then wipe off the surface moisture with a wring wet towel. Then weigh it and mark it *m*_2_. The water absorption of SFLAs is calculated according to Equation (2), and the calculation accuracy is 0.1%.

(2) $$W=\frac{m_{2}-m_{3}}{m_{3}}\times 100\%$$

where *W* is water absorption at 1 h (%); *m*_2_ is the mass of the soaked sample (g); *m*_3_ is the mass of the dried sample (g).

Apparent density test: based on the water absorption test of the SFLAs, the weighed sample (*m*_2_) was poured into a 1000 mL measuring glass, and then inject 500 mL clean water into the glass, and read the water level of the measuring cylinder quickly, regarded it as *V*_2_. The apparent density was calculated according to Equation (3), and the calculation was accurate to 1 kg/m^3^.

(3) $$\rho =\frac{m_{2}\times 1000}{V_{2}-500}$$

where *ρ* is the apparent density (kg/m^3^); *m*_2_ is the mass of the soaked sample (g); *V*_2_ is the total volume of the sample and water (mL).

Cylinder compressive strength test: take the 3 L samples and loaded into a standard pressure cylinder, then placed them on a vibration table to shake and smooth the surface so that the lower scale of the stamping die is aligned with the upper edge of the guide cylinder. Using DYE-2000 digital pressure testing machine. When the stamping die is pressed into a depth of 20 mm, the pressure value *p*_1_ is recorded. According to Equation (4), the cylinder compressive strength can be calculated with the calculation, and the calculation was accurate to 0.1 MPa.

(4) $$f=\frac{p_{1}+p_{2}}{F}$$

where *f* is cylinder compressive strength (MPa); *p*_1_ is the pressure value when the depth of pressing is 20 mm (N); *p*_2_ is the pressure value by the mass of the stamping die (N); *F* is the area of pressure bearing surface (mm^2^).

#### 2.4.3. Mineral Phases Analyzes of Binder Materials and RM-Based SFLAs

Based on the compressive strength test, binder materials and RM-based SFLAs were treated with absolute ethyl alcohol to terminate the hydration. The mineral phases of binder materials and RM-based SFLAs were identified via X-ray diffraction (XRD) with tube current and tube voltage at 30 mA, 40 kV; and a scanning speed of 8° min^–1^ over a range of 10–80°. XRD data was performed using the software to quantitatively analyze the phases.

### 2.5. Microstructure of Binder Materials and RM-Based SFLAs

In order to further study the microscopic process of hydration of RM-based SFLAs, SEM was used to test the microstructure of RM-based SFLAs and binder materials hydrated until 28 days.

### 2.6. Heavy Metal Leaching Test of RM-Based SFLAs

**Figure 4:**
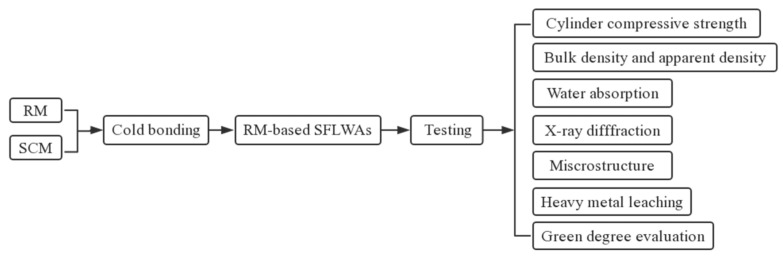
The experimental program of RM-based SFLAs.

The standard leaching test HJ 557-2010 was undertaken to determine the potential release of heavy metals from RM-based SFLAs. The leaching procedure involves the extraction of the solids with deionized water at the L/S ratio of 10 L·kg^−1^. The liquid was oscillated for 8 h and rest for 16 h, then the liquid was centrifuged 20 min at a speed of 3000 rpm. The liquid was separated with a 0.45mm pore membrane, the sample was refrigerated to below zero to 4 °C and stored for the test according to the requirements for analysis of the object under the test method. Finally, 8 elements including Mn, Cr, Ni, Cu, Zn, As, Cd, and Pb were analyzed by Optima 7000DV inductively coupled plasma optical emission spectrometry (ICP-OES), PerkinElmer Optima. Figure 4 shows the complete experimental program of RM-based SFLAs.

## 3. Results and Discussion

### 3.1. Properties of Solid Waste-Based Binder Materials

The physical and mechanical performance of the binder materials and Portland cement labeled 42.5R were tested and the test results are shown in Table 6. The early strength of binder material is obviously higher than ordinary Portland cement. In particular, they had a particularly higher at 1 d and 3 d hydration, which will be helpful to ensure the early strength of RM based on SFLAs. In addition, the initial and final setting times of binder materials were 40 min and 75 min, respectively. The binder materials set much faster than the Portland cement, which will contribute to powder materials cement together more quickly, shorten the pelletizing time and improve the pelletizing efficiency in the preparation of SFLAs.

**Figure 5:**
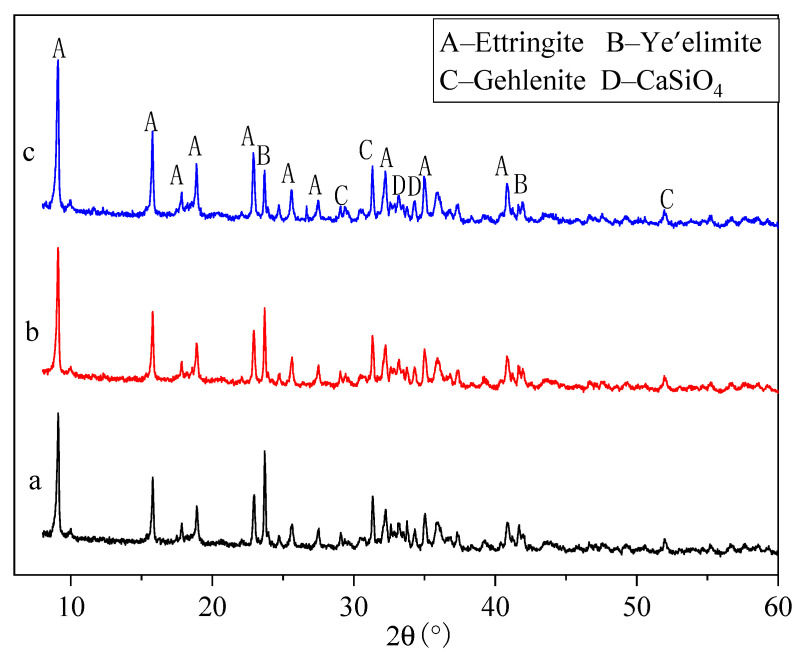
XRD patterns of hydrated binder materials (**a**–hydration 1 d, **b**–hydration 3 d, **c**–hydration 28 d).

The hydration reaction of binder materials was conducted at room temperature and pressure, it can be used for the production of RM-based SFLAs without autoclave curing. Figure 5 shows the XRD patterns of binder materials at different hydration ages. The main mineral phases of binder materials are: The XRD results showed that the main mineral phases of binder materials were ettringite (3CaO·Al_2_O_3_·3CaSO_4_·32H_2_O, AFT), unreacted ye’elimite (Ca_4_Al_6_O_12_SO_4__,_ C_4_A_3_S), gehlenite (Ca_2_Al_2_SiO_7_), and dicalcium silicate (Ca_2_SiO_4_, C_2_S). This is consistent with the research results of Yao et al. [33] that with the increase of hydration age, ettringite forms in large quantities and becomes the main crystalline phase of binder materials. As the hydration process progresses, the unreacted ye’elimite were gradually transformed into ettringite through the hydration, which guarantees the early strength of binder materials. Since hydration of C_2_S progresses relatively slowly and exerts a great influence on the strength at 28 d and later, C_2_S can ensure an increase in the later strength of binder materials.

### 3.2. Physical Performance of RM-Based SFLAs

#### 3.2.1. Particle Size Distribution

**Figure 6:**
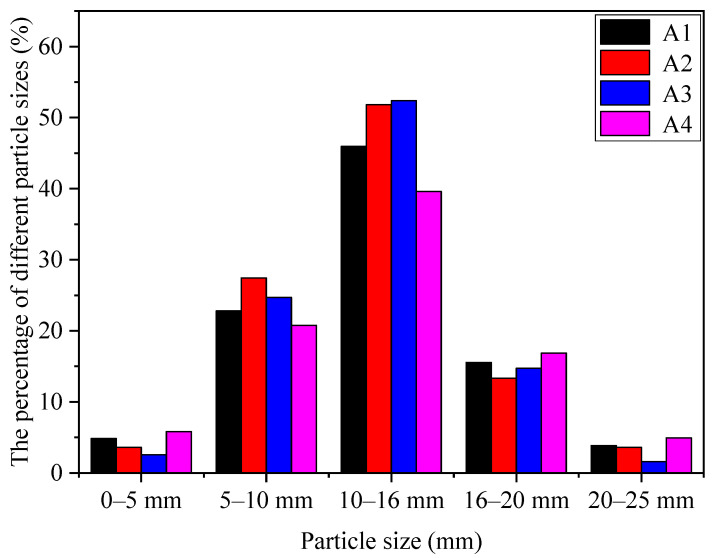
Particle size distribution of RM-based SFLAs.

Figure 6 shows the particle size distribution of RM-based SFLAs. The particle size of RM-based SFLAs was mainly centered in three ranges at 5–10 mm, 10–16 mm, and 16–20 mm. The proportion of samples with a particle size grade of 5–20 mm was 84.31%, 92.6%, 91.83%, and 77.25%, respectively. As the matching of binder materials increased, the pelletizing efficiency first increased and then decreased. The matching of binder materials is 20%, its cementing property is insufficient, resulting in a low pelletizing rate. The matching of binder materials is 50%, its reaction with water is too quick, the samples had a rough surface and uneven particle size. The results indicate that the preparation of RM-based SFLAs with 30% and 40% matching of binder materials was better than others. The particle sizes of A1, A2, A3, and A4 were all between 5–25 mm, meet the requirements of artificial light aggregate particle size grades in the GB/T 17431.1-2010. In this study, the RM-based SFLAs with particle sizes in the range of 5–10 mm, 10–16 mm, and 16–20 mm was collected for further study.

#### 3.2.2. Bulk Density and Apparent Density

**Figure 7:**
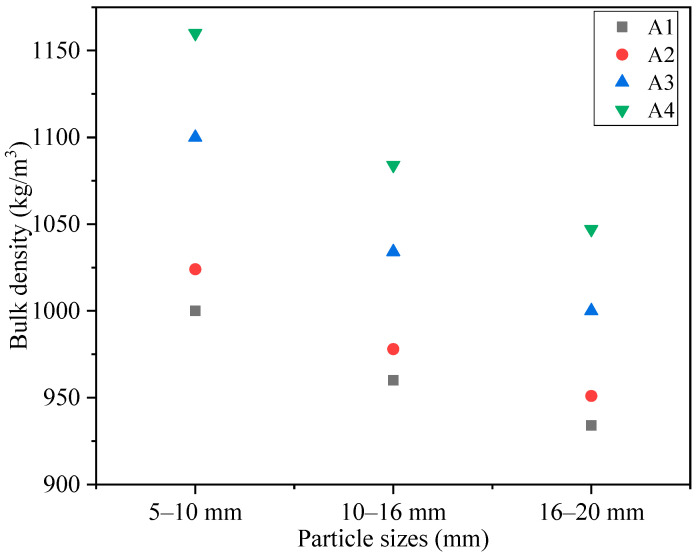
The bulk density of SFLAs with different particle sizes (kg·m^−3^).

Figure 7 depictes the influence of binder material matching and particle size on the bulk density of RM based on SFLAs. The bulk densities of 5–10 mm,10–16 mm and 16–20 mm were 1000, 960, and 934 kg·m^−3^ for A1; 1024, 978, and 951 kg·m^−3^ for A2; 1100, 1034, and 1000 kg·m^−3^ for A3; and 1160, 1084, and 1047 kg·m^−3^ for A4, respectively. The bulk density is mainly related to the mutual filling state of aggregates in the steel cylinder, the smaller the particle size, the larger the coordination number, the smaller the porosity, and the higher the bulk density. With the matching of binder materials increasing, the compactness of SFLAs increases, this is the essential reason that the bulk densities of the SFLAs increase. GB/T 17431.1-2010 defines the density grade of artificial lightweight aggregate as 1000 density grade. When the matching of binder materials was 20%, all A1 of 5–20 mm samples meet the national standard requirements for lightweight aggregates. When the binder content was 30%, A2 of 10–20 mm met the national standard requirements. Also, only A3 of 16–20 mm samples met when the matching of binder materials was 40%. However, when the matching of binder materials was 50%, none of A4 samples met the national standard requirements for lightweight aggregates. This indicates that the binder content of 20% and 30% is better to prepare RM based on SFLAs.

Table 7 shows the relationship between the binder content and apparent density. As the matching of binder materials increased, the apparent density of the SFLAs increased. The apparent density is closely related to the pore structure. The minerals generated by hydration filled the pores inside the SFLAs, which improved the pore structure of the system and increased the compactness. However, the apparent density of RM based on SFLAs with different particle sizes did not change significantly because the apparent density was the ratio of mass to volume, which was not related to the size of particle size. The difference may be due to the error caused by the drainage method to measure the volume of the aggregate.

#### 3.2.3. Cylinder Compressive Strength

**Figure 8:**
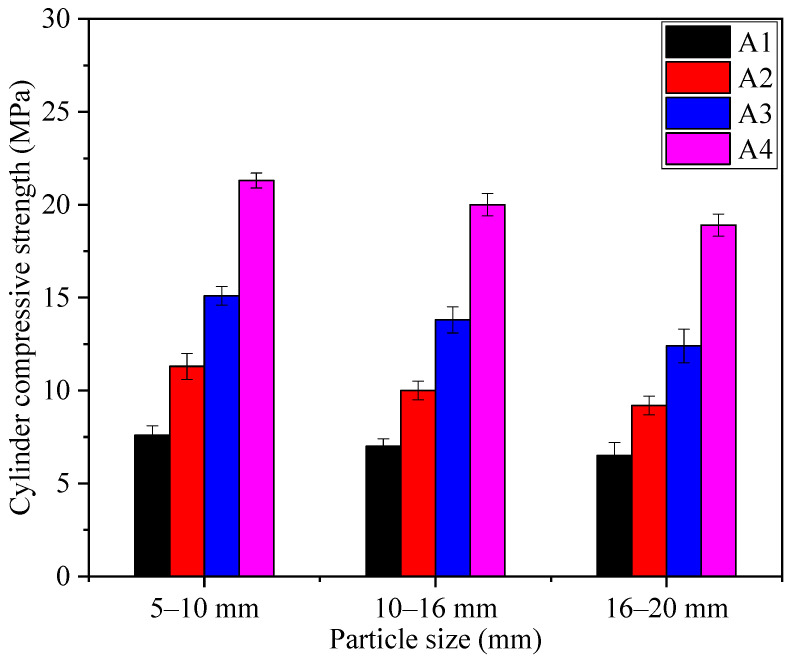
The influence of particle sizes on cylinder compressive strength.

The cylinder compressive strength is an important parameter to evaluate the mechanical properties of SFLAs. The SFLAs with particle size grades of 5–10, 10–16, and 16–20 mm were selected from the samples of A1, A2, A3, and A4 to study the effect of particle size on cylinder compressive strength. The test results are shown in Figure 8. The cylinder compressive strength of A1, A2, A3, and A4 with a particle size of 5–10 mm were 7.6, 11.3, 15.1, and 21.3 MPa, respectively, and the cylinder compressive strength for these samples with a particle size grade of 16–20 mm were 6.5, 9.2, 12.4, and 18.9 MPa. The cylinder compressive strength of the SFLAs increased significantly with the increase of binder materials matching. Thus, the binder materials matching is very important for optimizing the mechanical properties of the SFLAs. The cylinder compressive strength declined as the particle size grade increased, this difference is due to the different effects of mechanical force and capillary force on different particle size grades.

**Figure 9:**
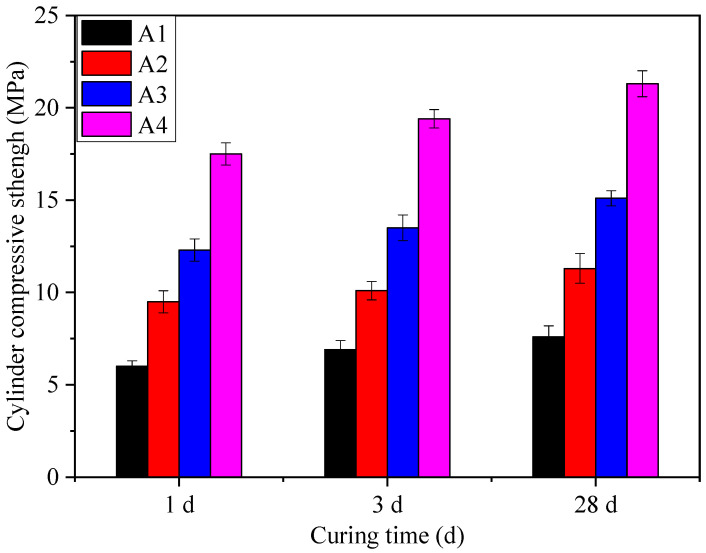
The influence of curing ages on cylinder compressive strength.

In order to study the influence of curing age on the cylinder compressive strength of RM based on SFLAs, the cylinder compressive strength of RM based on SFLAs with particle size of 10–16 mm at different curing ages were tested, and the results were shown in Figure 9. When the matching of binder materials was 20%, 30%, 40%, and 50%, the cylinder compressive strength of the RM-based SFLAs was 7.6, 11.3, 15.1, and 21.3 MPa after natural curing for 28 d, respectively, all of which meet and exceed the standard index requirement for high-strength artificial lightweight aggregates, which requires the cylinder compressive strength is more than 6.5 MPa. The results show that the cylinder compressive strength of samples increases with increases of binder materials matching, when the binder materials matching is fixed, longer curing time leads to higher cylinder compressive strength. With the increase of curing age, the amount of ettringite produced by hydration of binder materials increases, which fills the internal pore structure of SFLAs, and the internal structure is more compact. This is the essential reason for the increase in cylinder compressive strength.

#### 3.2.4. Water Absorption

**Figure 10:**
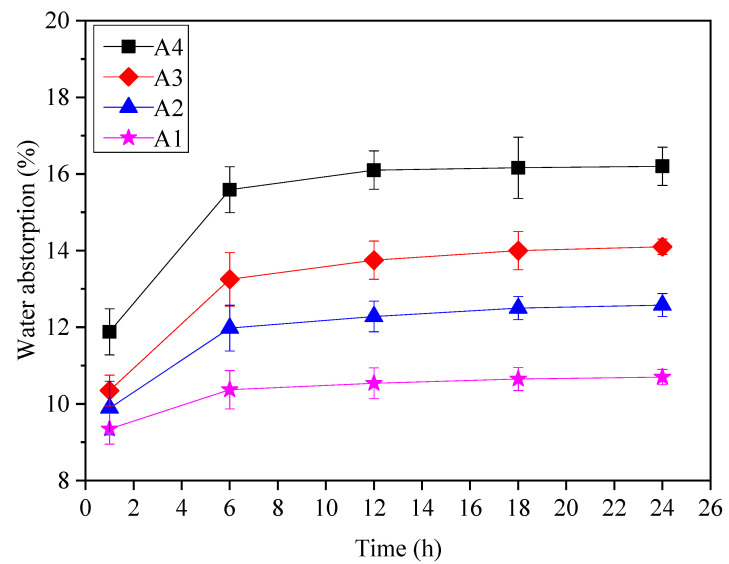
Water absorption of RM-based SFLAs.

The quality of lightweight aggregate used in building materials has an important relationship with water absorption, which will affect the strength of lightweight aggregate. Figure 10 shows the water absorption of RM-based SFLAs with different binder content. The water absorption of different types of RM-based SFLAs shows roughly the same trend. In the early stage, as the water inside the aggregate has not reached the saturation state, the water absorption rate increases with the increase of time. When the aggregate is full of water, the water absorption reaches a stable value. When the content of the binder is 20% (A1), most of the aggregate is red mud, which has poor cementation and high water absorption. With the increase of binder content, the cementitious property of binder hydration is enhanced when the water penetrates the interior of the RM-based SFLAs, it is not easy to produce a mud slurry reaction, and its water absorption is reduced. According to GB/T 17431.1-2010, for artificial light aggregate with a density grade of 600–1200, the water absorption for 1 h is less than 10%. The water absorption by the samples in group A2, A3, and A4 was, respectively, 10.35%, 9.89%, and 9.35%. Therefore, when the material matching of the binder is more than 20%, the water absorption of the RM-based SFLAs at 1 h meet the national standard requirements.

### 3.3. XRD Analysis of RM-Based SFLAs

**Figure 11:**
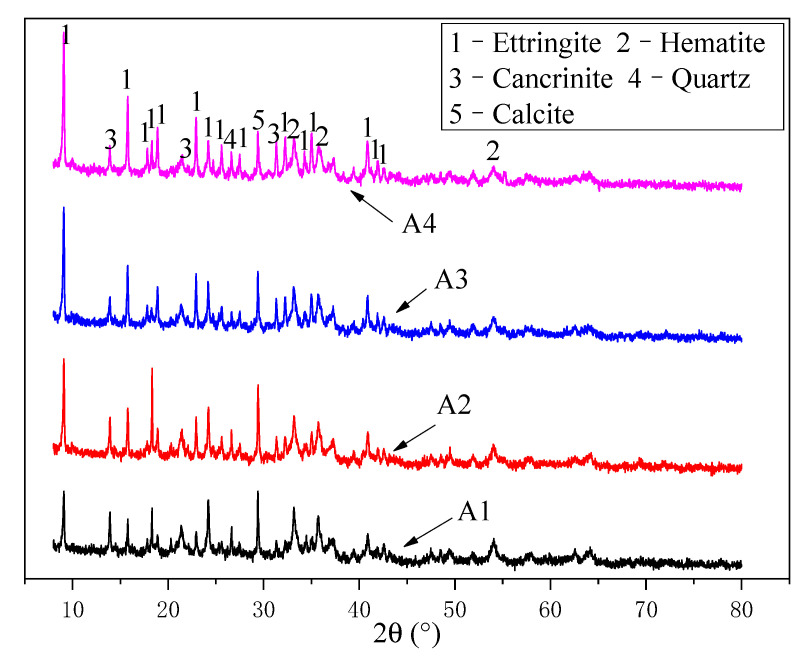
XRD patterns of RM-based SFLAs at 28 d hydration.

Figure 11 displays the XRD pattern of the RM-based SFLAs with different material matching of binder after natural curing for 28 d. The main mineral phases of SFLAs was AFt, hematite, cancrinite (Na_6_Ca_2_Al_6_Si_6_O_24_(CO_3_)_2_·2H_2_O), quartz (SiO_2_), and calcite (CaCO_3_). The cementitious connection between RM and binder governs the structural strength of SFLAs. At the early stage of the hydration reaction, the AFT phase generated is the main contributor to the higher mechanical properties of SFLAs. At the later stage of the hydration reaction, with the further formation of the AFt phase, the mechanical properties of SFLAs at the later stage are guaranteed, which also verifies the potential of binder can afford good engineering performance to RM-based SFLAs.

### 3.4. Microstructure. Analysis of RM-Based SFLAs

**Figure 12:**
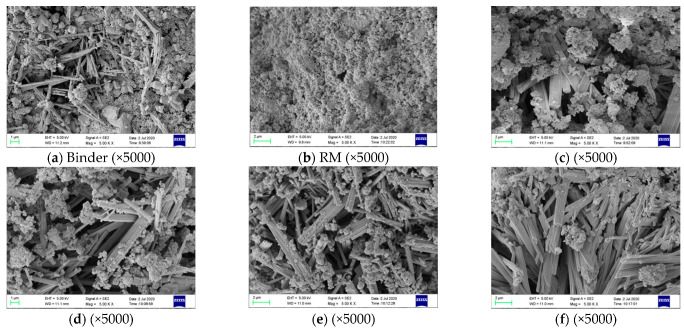
SEM images of binder, RM, and RM-based SFLAs.

The SEM images of binder material, RM, and RM-based SFLAs are presented in Figure 12. Figure 12a presents the microstructure of binder hydration after 28 d, ettringite crystals are arranged in regular columnar form, the gaps between ettringite crystals were filled with gel produced by hydration and minerals not completely hydrated to obtain a denser structure, which gives the binder specimen a good macroscopic mechanical strength. Figure 12b presents the microstructure of the RM itself, RM samples were distributed in granular form and are interconnected as a whole. Figure 12c–f, respectively, present the microstructure of RM-based SFLAs with different material matching of binder after natural curing for 28 d, it can be seen that this columnar ettringite lapped to form a skeleton structure, and gel by hydration and unhydrated red mud particles cemented or filled in the skeleton. Besides, with the increase of binder matching, the amount of ettringite increased significantly, which was consistent with the results of XRD analysis, and also well explained the improvement of SFLAs strength.

### 3.5. Leaching Behavior of RM-Based SFLAs

To determine the potential environmental impact of RM-based SFLAs, the standard leaching test HJ 557-2010 [34] was performed on the representative samples using ICP. Table 8 shows the leaching concentration of heavy metal ions in RM-based SFLAs at different curing ages. The concentration of Mn, Cr, Ni, Cu, Zn, As, Cd and Pb leached from all samples was very low, indicating that the leaching of heavy metal ions such as Ni, Cu, Zn, Mn, Cd, As, and Pb was not affected by the hydration of binder. However, Cr leaching was more significant, and the concentration of leached Cr decreased with the curing age. Further, when the matching of the binder was higher, and the curing age was longer, the concentration of leached Cr was lower. According to the GB/T 5085.3-2007 [35] “Hazardous Waste Identification Standard”, the total concentration of leached Cr should be less than 15 mg·L^−1^. In this study, the highest concentration of Cr leached from the samples was 0.47 mg·L^−1^, which is clearly within the limit of the leaching toxicity identification standard. Studies have shown that ettringite produced by hydration of binder can effectively reduce the Cr migration capacity and has a good stabilizing effect on Cr [36,37]. The data show that the concentration of leached Cr decreased significantly with time-based on a comparison of the 1 d and 28 d samples. The amount of ettringite increases with the progress of binder hydration (age) for RM-based SFLAs, and the stabilizing effect of ettringite restricts the migration of Cr in RM-based SFLAs, thus reducing the concentration of Cr leached from RM-based SFLAs. This is consistent with the results of previous studies where ettringite showed a good stabilizing effect on Cr.

### 3.6. Comparison between RM-Based SFLAs and Conventional LAs

Binder is an essential element in the pelletizing process, especially when a raw material with little or no cementitious properties is pelletized. In order to further illustrate the value and advantages of RM-based SFLAs, the comparison between RM-based SFLAs and conventional LAs was made in terms of particle size, raw materials, binder, additive, bulking density (BD), water absorption (WAB), and cylinder compression strength (CCB). As can be seen in Table 9, Portland cement (CEM) is widely used as the binder, Polypropylene fiber (PPF), crumb rubber (CR) as the admixture, hydrogen peroxide (HP) as the foaming agent, Na_2_SiO_3_, NaOH as the activator to activate the potential activity of fly ash, to further produce LAs meeting specific performance requirements [38,39,40,41,42]. This article proposes for the first time that solid waste-based sulphoaluminate cement was used as the binder and red mud was used as raw material to realize the preparation of RM-based SFLAs through a cold bonding process, without the use of any additives, and the utilization rate of RM up to 80%. Since the raw materials for preparing the RM-based SFLAs were entirely from industrial solid waste, its preparation cost is greatly reduced. The heavy metal leaching index meets the requirements of heavy metal leaching standards for building materials, and will not cause secondary pollution to the environment. With the same particle size grade, the bulk density (BD) of RM-based SFLAs is slightly higher than conventional LAs, but it also meets the requirements of lightweight coarse aggregate. The water absorption (WAB) of RM-based SFLAs is much lower than conventional LAs, which will facilitate the preparation of lightweight aggregate concrete, and the WAB of lightweight aggregate determines the performance of lightweight aggregate concrete to a certain extent. Under the same curing conditions, the cylinder compressive strength (CCS) of RM-based SFLAs is significantly higher than conventional LAs. Only under autoclaved curing conditions, the CCS of conventional LAs is slightly higher than that RM based on SFLAs, indicating that RM-based SFLAs have absolute advantages and value in mechanical properties. In summary, the RM-based SFLAs is low-cost, environmentally friendly, and good-performance LAs. Converting RM into RM-based SFLAs as building materials has a very broad market prospect.

## 4. Conclusions

In this study, a combination of RM and binder form whole solid waste was innovatively used to produce sintering-free lightweight aggregates (SFLAs). This paper mainly studied the preparation method, and the influence of the material matching on the physical and chemical properties of RM based on SFLAs, evaluated the leaching of heavy metals from RM-based SFLAs, and finally, the raw materials, binders, additives and physical properties of RM based on SFLAs, and conventional LAs were compared and analyzed. The following conclusions can be drawn:

(1) It is feasible to use a high proportion of red mud to prepare RM based on SFLAs by using the pelletizing technique, and solid waste-based binder can be used as an alternative cementitious material, having excellent compatibility with RM in preparation of RM based on SFLAs. Moreover, the present study demonstrates a new SFLAs preparation method based totally on solid wastes.
(2) The columnar ettringite is lapped to form a skeleton structure, and gel by hydration and unhydrated red mud particles cemented or filled in the skeleton. This structure increases the compactness of SFLAs, which is the fundamental reason for the high strength of RM based on SFLAs. Therefore, the binder can be used as a substitute for Portland cement to produce SFLAs.
(3) The raw materials for the preparation of SFLAs were all from industrial solid waste, which greatly reduced the preparation cost of SFLAs and environmental problems caused by the massive storage of RM. The optimal matching of binder/RM was 3:7, and the prepared SFLAs had a 28 d cylinder compressive strength of 11.3 MPa, bulk density of 900–1000 kg·m^−3^, and water absorption of less than 10%. It provides a theoretical basis for the preparation and application of red mud-based aggregate, which can be used for the preparation of lightweight aggregate concrete and roadbed fillers, etc., with broad application prospects.
(4) The concentration of heavy metals leached from SFLAs is lower than the toxic leaching standard for building materials. Besides, as the hydration age increases, the number of chromium ions leached from the red mud aggregates will decrease, because ettringite produced by hydration of the binder material has a good stabilizing effect on Cr and demonstrates good environmental compatibility. Therefore, RM-based SFLAs have good environmental acceptable, and will not cause secondary pollution to the environment.
(5) Compared with conventional LAs, RM based on SFLAs has the advantages of a simple preparation process, lower cost, and better performance than conventional LAs, which further proves that the red mud combined with binder material to prepare red mud aggregate is an effective way to recycle red mud.

## Figures and Tables

**Table 1:** Basic physical properties of red mud.

Size Distribution (mm)	Dry Density (kN/m^3^)	Specific Gravity (kN/m^3^)	pH	Water Content (%)	Void Ratio	BET (m^2^/g)
0.08–2.25	7.54	2.8	12.3	35.3	2.64	20.24

**Table 2:** Chemical composition of raw materials (wt. %).

	CaO	SiO_2_	Al_2_O_3_	Fe_2_O_3_	SO_3_	MgO	TiO_2_	R_2_O ^a^	LOI ^b^
RM	6.61	19.23	22.33	31.19	0.64	0.93	4.13	1.6	12.93
FGD gypsum	34.52	0.81	1.08	0.15	44.47	0.71	0.03	-	16.99
Aluminum dust	4.20	6.58	60.98	0.71	0.94	6.26	0.78	1.80	12.19
Carbide slag	65.12	2.52	1.50	0.29	0.51	0.20	0.02	0.02	29.63

^a^ Alkaline oxide (K_2_O, Na_2_O); ^b^ Loss at ignition of 950 °C.

**Table 3:** Compositions and parameters of the raw material (wt. %).

C_m_	P	N	FGD Gypsum	RM	Aluminum Dust	Carbide Slag
0.90	1.9	3.4	21.92	22.98	23.43	31.67
0.95	1.9	3.4	21.87	20.86	22.31	34.96
1.00	1.9	3.4	21.27	20.29	21.20	36.74
1.05	1.9	3.4	20.69	19.74	21.11	38.46

**Table 4:** Chemical composition of binder materials (wt. %).

	CaO	SiO_2_	Al_2_O_3_	Fe_2_O_3_	SO_3_	MgO	TiO_2_	R_2_O ^a^	LOI ^b^
Proportion	36.70	8.31	26.03	9.31	14.53	2.83	1.41	0.36	0.76

^a^ Alkaline oxide (K_2_O, Na_2_O); ^b^ Loss at ignition of 950 °C.

**Table 5:** The material matching and pelletizing process parameters.

	RM (%)	Binder (%)	Water (%)	Time (min)	Diameter (mm)	Angle (°)	Critical Revolutions (rpm)
A1	80	20	23	14	50	45	33
A2	70	30	23	14	50	45	33
A3	60	40	23	14	50	45	33
A4	50	50	23	14	50	45	33

**Table 6:** Physical and mechanical performance of binder materials and Portland cement.

	Mortar Compressive Strength (MPa)			Flexural Strength (MPa)			Setting Time (min)	
	1 d	3 d	28 d	1 d	3 d	28 d	initial	final
Binder material	38.3	49.4	58.7	6.5	7.0	8.7	40	75
Portland cement	16.2	24.1	43.7	5.7	6.3	8.2	190	278

**Table 7:** The apparent density of SFLAs with different particle sizes (kg·m^−3^).

Particle Size (mm)	A1	A2	A3	A4
5–10 mm	1922	1977	2033	2076
10–16 mm	1925	1974	2035	2074
16–20 mm	1919	1978	2036	2079

**Table 8:** Heavy metals leaching concentration of RM-based SFLAs at different curing ages (mg/L).

Samples	Curing Ages	Ni	Cu	Zn	Mn	Cr	Cd	As	Pb
RM	-	0.049	0.110	0.077	0.069	1.016	0.076	0.105	0.082
Binder	-	0.118	0.069	0.290	0.148	0.195	0.021	0.044	0.016
A1	1 d	0.005	0.016	0.053	0.053	0.470	0.011	0.025	0.025
	3 d	0.06	0.015	0.053	0.044	0.362	0.011	0.022	0.025
	28 d	0.005	0.013	0.054	0.043	0.196	0.011	0.019	0.024
A2	1 d	0.005	0.014	0.055	0.054	0.402	0.011	0.026	0.025
	3 d	0.006	0.024	0.056	0.041	0.289	0.011	0.021	0.023
	28 d	0.005	0.013	0.056	0.039	0.137	0.010	0.018	0.021
A3	1 d	0.005	0.013	0.056	0.054	0.291	0.011	0.036	0.026
	3 d	0.003	0.012	0.057	0.040	0.183	0.010	0.027	0.025
	28 d	0.002	0.012	0.057	0.038	0.104	0.010	0.017	0.024
A4	1 d	0.001	0.011	0.057	0.054	0.175	0.010	0.034	0.029
	3 d	ND	0.011	0.058	0.039	0.106	0.010	0.031	0.028
	28 d	ND	0.011	0.058	0.038	0.083	0.010	0.028	0.027
Leaching limit value		<5	<100	<100	<100	<15	<100	<5	<5

**Table 9:** Physical properties of RM-based SFLAs and conventional lightweight aggregates (LAs).

Reference	Size (mm)	Raw Material	Binder	Water Content (wt.%)	Additive	BD (kg/m^3^)	WAR (wt.%)	CCS (MPa)	Method of Curing
In this work	10–16	Red Mud	Solid waste-based Binder	23	No additive	900–1000	9.35–12	11.3	At room tempera-ture
[37]	4–10	Class-F FA, GGBFA	PC and Na_2_SiO_3_	-	HP and NaOH	765–915	13.7–25.6	4.73–8	RH 60% at 23 °C
[38]	4–16	Class-F FA	PC CEM I 42.5 R	23–27	PPF and CR	1000	24–28	3.53–4.19	RH 80% at 20 °C
[39]	10–12.5	Class-F FA	Bentonite	25	NaOH	950	16.39	10.22–14.51	Hot air oven at 100 °C
[40]	4.75–19	Class-F FA	PC CEM I 42.5 R	22–25	-	789	-	3.7	RH 70% at 25 °C
[41]	4.76–12.7	Class-F FA	PC CEM I	-	-	857–972	20–35	6–8.57	-

## Data Availability

Data is contained within the article or supplementary material.
